# Community-supported self-administered tuberculosis treatment combined with active tuberculosis screening: a pilot experience in Conakry, Guinea

**DOI:** 10.1080/16549716.2023.2262134

**Published:** 2023-10-06

**Authors:** Souleymane Hassane-Harouna, Tinne Gils, Tom Decroo, Nimer Ortuño-Gutiérrez, Alexandre Delamou, Gba-Foromo Cherif, Lansana Mady Camara, Leen Rigouts, Bouke Catherine de Jong

**Affiliations:** a Tuberculosis department, Damien Foundation, Conakry, Guinea; b Unit of HIV & Co-infections, Department of Clinical Sciences, Institute of Tropical Medicine, Antwerp, Belgium; c Global Health Institute, University of Antwerp, Antwerp, Belgium; d Unit of Research, Damien Foundation, Brussels, Belgium; e Unit of Research, Public Health Department, Gamal Abdel Nasser University, Conakry, Guinea; f Unit of Mycobacteriology, Department of Biomedical Sciences, Institute of Tropical Medicine, Antwerp, Belgium

**Keywords:** SAT, decentralized treatment, TB-case finding, TPT, differentiated TB care

## Abstract

Directly observed treatment (DOT) for tuberculosis (TB) is recommended by the World Health Organization. However, DOT does not always meet patients’ preferences, burdens health facilities, and is hard to implement in settings where access to healthcare services is regularly interrupted. A model addressing these limitations of DOT is community-supported self-administered treatment (CS-SAT), in which patients who self-administer TB treatment receive regular visits from community members. Guinea is a country with a high TB burden, recurrent epidemics, and periodic socio-political unrest. We piloted a CS-SAT model for drug-susceptible TB patients in Conakry, led by community volunteers, who also conducted active TB case finding among household contacts and referrals for isoniazid preventive treatment (IPT) in children below 5 years old. We aimed to assess TB treatment outcomes of patients on CS-SAT and describe the number of patients identified with TB case finding and IPT provision. Prospectively enrolled bacteriologically confirmed TB patients, presenting to two facilities, received monthly TB medication. Community volunteers performed bi-weekly (initiation phase) and later monthly (continuation phase) home visits to verify treatment adherence, screen household contacts for TB, and assess IPT uptake in children under five. Among 359 enrolled TB patients, 237 (66.0%) were male, and 37 (10.3%) were HIV-positive. Three hundred forty (94.7%) participants had treatment success, seven (1.9%) died, seven (1.9%) experienced treatment failure, and five (1.4%) were lost-to-follow-up. Among 1585 household contacts screened for TB, 26 (1.6%) had TB symptoms, of whom five (19.2%) were diagnosed with pulmonary TB. IPT referral was done for 376 children from 198 households. In a challenging setting, where DOT is often not feasible, CS-SAT led to successful TB treatment outcomes and created an opportunity for active TB case finding and IPT referral. We recommend the Guinean CS-SAT model for implementation in similar settings.

## Introduction

Tuberculosis (TB) is one of the leading causes of death from a single infectious agent. Global treatment success was 86% in the 2020 cohort with rifampicin-susceptible TB [1]. The World Health Organization (WHO) recommended directly observed treatment (DOT) short-course strategy in 1994 including 1) government commitment, 2) high-quality sputum smear microscopy, 3) directly observed use of standardised treatment by health staff at a facility for at least the initial 2 months, 4) uninterrupted supply of short-course anti-TB drugs and 5) a standardised TB outcome recording and reporting system [2,3].

In low-income countries, daily health facility-based DOT is challenging, as it requires substantial health service financial and human resources, especially in high-volume health facilities. Daily health facility-based DOT also burdens patients and their families through direct costs (e.g. transportation fees) and indirect expenses related to the long time spent in health facilities [4,5]. Moreover, the socio-political and health-related contexts often hinder health service continuity. This is the case in Guinea, where the TB incidence is high (175/100,000 in 2021) [6]. The country faces recurrent periods of socio-political unrest. The Ebola virus outbreak also gravely affected the already weakened health system [7]. Public health and social measures to control virus circulation during the Ebola outbreak highlighted the limitations of daily health facility-based DOT [8,9].

Alternative TB treatment delivery models exist, aiming to address DOT limitations and improve treatment outcomes. Community-supported self-administered treatment (CS-SAT) of TB medication is a model in which patients are not supervised daily but receive regular support visits in their community [10]. Review papers evaluating the effectiveness of TB treatment models document contrasting results depending on the model definition, inclusion criteria, and outcomes measured. In one meta-analysis, McKay et al. found no statistically significant differences in rifampicin-susceptible TB treatment outcomes between SAT and DOT [11], while Tian et al. found community-delivered DOT (but not facility-based) to be more effective compared to SAT [12]. Outcomes after CS-SAT versus DOT in rifampicin-resistant TB did not differ in a South African study [10].

To our knowledge, no studies have documented outcomes of CS-SAT or other DOT alternatives in West and Central Africa, where healthcare access is often interrupted. Evidence on using community-based TB treatment models to deliver recommended TB screening in household contacts is limited. We aimed to evaluate treatment outcomes after CS-SAT in rifampicin-susceptible TB and describe the benefits of combining household visits with active TB screening of household members and referral for isoniazid preventive therapy (IPT) referral in children under 5 years old.

## Methods

We prospectively enrolled bacteriologically confirmed pulmonary TB patients treated for drug-susceptible TB between October 2018 and December 2019. Two years after the end of the Ebola epidemic, the health system was still recovering in terms of resources, including staff, and patients’ trust in the health sector.

Smear microscopy with Ziehl Neelsen staining was used for TB diagnosis. Guinea’s estimated TB incidence rate was 175/100,000 inhabitants in 2020 [6], and 50% of cases are present in the capital Conakry. The study was conducted in two high-patient burden TB centres supported by the international organisation Damien Foundation in Ratoma and Matoto districts, where 79% of the city’s 1,950,000 inhabitants live [13]. Guinean guidelines prescribe facility-based daily DOT during the intensive phase and weekly drug supply in the continuation phase. For confirmed TB cases, household contact TB screening and IPT initiation among all children under 5 years old, after excluding active TB, are recommended [14].

In a meta-analysis of eight studies, community-based observed treatment reached 78.4% treatment success [5]. To estimate a proportion of 78% treatment success (alpha error = 0.05, power = 80%), allowing a minimum proportion of 70%, 270 participants needed to be enrolled. The study intervention consisted of home-based self-administered TB treatment, supported by a community volunteer, and household active TB case finding. One trained community volunteer was assigned per TB centre. The two volunteers received a monthly incentive, a smartphone with Open Data Kit (ODK), an open-source application, and a motorcycle for home visits. Around 25 pulmonary TB patients were diagnosed monthly in each TB centre. When TB was diagnosed, the nurse and community volunteer explained the study to the patient/parent and sought their consent (assent with guardian consent in case of minors) to participate (or for their child to participate) in the study, including to accept the home visits. After consent/assent, a monthly drug supply was provided to the patient or parent (in case of minors). Participants were visited by the community volunteer at home bi-weekly in the intensive phase and monthly during the continuation phase. During home visits, community volunteers monitored treatment adherence by pill count, investigated the occurrence of adverse events, recorded complaints, and conducted TB screening among household contacts. Household members with a cough for over 2 weeks, fever, or weight loss were asked to provide a sputum sample at the household. Samples were transported to the TB centre, where smear microscopy was performed. Children under 5 years old present at the household, who were not yet enrolled on IPT, were referred to the TB centre for investigation of active TB, and if TB was excluded, they were initiated on IPT. In between home visits, the community volunteer conducted one phone call to encourage adherence and inquire about adverse events. Patients visited the facility at months two, five, and six for sputum smear microscopy. TB nurses reported results in TB registers and individual patient treatment files, while community volunteers recorded information in ODK. Case report forms and outcome definitions followed WHO guidelines [15]. We presented descriptive statistics and calculated 95% confidence intervals for the main outcomes with Stata version 16.01 (Stata Corp, USA). The Comité National d’Ethique pour la Recherche en Santé of the Republic of Guinea (N°015/CNERS/18) approved the study. All participants signed informed consent.

## Results

Overall, 94.7% [95% CI 91.9–96.8] patients experienced treatment success, and 5.3% [95% CI 3.2–8.1] had an adverse outcome (Table 1).

**Table 1.** Characteristics and treatment outcomes of TB patients (*N* = 359) receiving CS-SAT in Guinea, 2018–19.

Characteristics		n (%)
Gender	Female	122 (34.0)
	Male	237 (66.0)
Age (years, median [IQR])		29 [21–40]
Age (years, category)	≤25	150 (41.8)
	26–44	145 (40.4)
	≥45	64 (17.8)
HIV status	Negative	320 (89.1)
	Positive	37 (10.3)
	Unknown	2 (0.6)
**Outcomes**		
Treatment success		340 (94.7)
	Cured	339 (94.4)
	Completed	1 (0.3)
Adverse outcomes		19 (5.3)
	Died	7 (1.9)
	Treatment failure	7 (1.9)
	Lost-to-follow-up	5 (1.4)

IQR: interquartile range, CS-SAT: community-supported self-administered treatment, TB: tuberculosis.

**Figure 1.**
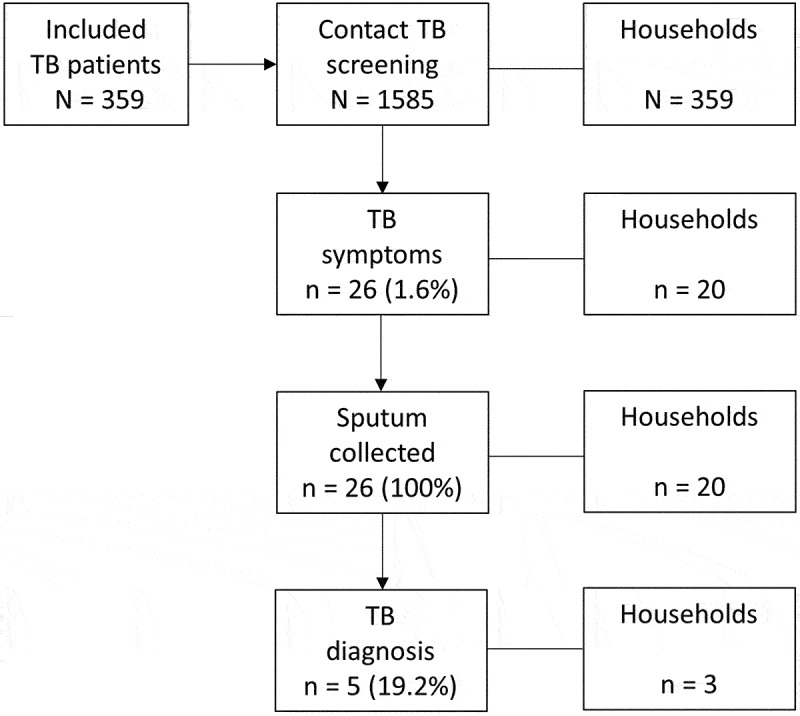
Flow chart of household TB screening during CS-SAT in Guinea, 2018–19.

In 359 households, 1585 contacts were screened (median: 4 [IQR: 3–6] contacts per patient) (Figure 1).

CS-SAT: community-supported self-administered treatment, TB: tuberculosis.

Five cases of with bacteriologically confirmed TB were identified in three households (two households with two cases, one with one case). The number needed to screen to diagnose one TB case was 317. In total, 376 children were identified for referral to receive IPT in households of 198 patients (median: 2 [IQR: 1–2] children per household).

## Discussion

We present results of CS-SAT in Guinea, contributing to the limited data on alternative TB treatment models from the West and Central African region. In Guinea, we achieved an excellent 94.7% [95% CI 91.9–96.8] treatment success in patients on CS-SAT, exceeding the National TB Programme’s (NTP) treatment success target of 87% for 2016–2020 and the 90% national treatment success reached in 2019 [9,16]. A 2019 review found no superiority of SAT over DOT for treatment completion, but both reached only 72% completion [5]. More recently, SAT achieved 91% treatment success versus 81% in family-supported DOT and 77% for DOT in Papua New Guinea [17].

The community volunteer support in our model is likely to have contributed to treatment success by stimulating adherence. Community visits were optimised as opportunities for household contact screening. Five TB cases were found among 1585 screened contacts, or a number needed to screen of 317 to find one case. The sensitivity of smear microscopy is limited so that we may have missed TB cases. Indeed, among 11 TB cases identified from 282 screened contacts in South Africa, only one was identified by smear microscopy and others by culture [18]. Our study also allowed for the referral of 376 eligible children for IPT initiation who the NTP had not yet identified. The community-volunteer contact during CS-SAT thus provided an opportunity to implement underutilised policy with already established benefits.

Our study has limitations. We did not include a control group on DOT nor did we assess the cost-effectiveness of our CS-SAT model. Damien Foundation provided motorcycles and maintenance costs, as well as monthly incentives for community volunteers, which could be costly for the NTP. However, review data show that TB treatment observed daily by community volunteers can be cost-effective [19]. At study completion, motorcycles were donated for routine programme activities. We did not perform post-treatment follow-up or assess patient satisfaction or quality of life.

Our study results already impacted policy. During the COVID-19 pandemic, the WHO recommended providing adequate supplies of TB medicines to all patients for home-based TB treatment to reduce the frequency of patients’ and caregivers’ contacts [20]. Our findings facilitated implementation of these TB treatment delivery flexibilities in Guinea. Since then, SAT is allowed on a case-by-case basis in the continuation phase. Future research on TB treatment models should include contexts where access to health is often impaired and assess patient preference. Newer treatment models, e.g. video-DOT, are probably interesting in these settings and should be evaluated [21,22].

CS-SAT was feasible and successfully treated susceptible pulmonary TB in Guinea, a challenging setting. Community visits yielded additional TB cases and children eligible for IPT through active case finding. CS-SAT implementation should be considered in Guinea and other settings, where continuity of care is regularly interrupted.

## Paper context

Daily health facility-based DOT for TB is still standard of care in many countries in West and Central Africa, where access to health facilities is often limited for patients, yet flexibilities for decentralised TB treatment such as CS-SAT, which have shown positive outcomes elsewhere, are not yet widely implemented in the region. We present the outcomes of CS-SAT, in combination with household TB case finding in Guinea, contributing to the limited evidence from the region.

## Data deposition

The data that support the findings of this study are openly available in figshare at https://doi.org/10.6084/m9.figshare.24211632.v1.
